# Gene Therapy for Cardiovascular and Cerebrovascular Disease: Mechanisms, Translational Barriers, and the Road Ahead

**DOI:** 10.3390/biomedicines14051142

**Published:** 2026-05-18

**Authors:** Zixu Liu, Ruiqi Liu, Ying Ying, Jing Nie

**Affiliations:** 1 Queen Mary College, Jiangxi Medical College, Nanchang University, Nanchang 330006, China; zixu.liu@se22.qmul.ac.uk (Z.L.); ruiqi.liu@se22.qmul.ac.uk (R.L.); 2 Jiangxi Provincial Key Laboratory of Prevention and Treatment of Infectious Diseases, Jiangxi Medical Centre for Critical Public Health Events, The First Affiliated Hospital, Jiangxi Medical College, Nanchang University, Nanchang 330052, China; yingying@ncu.edu.cn; 3 Department of Anatomy, School of Basic Medical Sciences, Jiangxi Medical College, Nanchang University, Nanchang 330006, China

**Keywords:** cardiovascular disease, cerebrovascular disease, gene therapy, cardiac arrhythmia, atherosclerosis, ischaemic stroke

## Abstract

Cardiovascular and cerebrovascular diseases, encompassing cardiac arrhythmias, atherosclerosis, and ischaemic stroke, remain the foremost causes of death and long-term disability globally. Despite improved outcomes with conventional therapy, substantial residual risk persists, providing the impetus for gene-based intervention. KCNQ1/KCNH2 suppression-and-replacement, SCN5A base editing, and structural protein restoration via PKP2 and TMEM43 have each demonstrated capacity to re-establish electrophysiological stability in arrhythmia models. For atherosclerosis, RNA-based agents, notably inclisiran, alongside in vivo editing strategies such as VERVE-101, offer durable lipid reduction and attenuation of vascular inflammation. In ischaemic stroke, cGAS–STING silencing, AAV-NeuroD1-mediated neuronal reprogramming, and delivery of neurotrophic factors, including VEGF and BDNF, extend the therapeutic window well beyond reperfusion. Collectively, these approaches position gene therapy as a meaningful complement to standard care, capable of addressing root molecular pathology rather than downstream consequences. This review synthesises current mechanistic understanding, translational obstacles, and emerging directions across these three disease domains, arguing that, delivery and safety challenges notwithstanding, gene therapy stands to substantially reshape how cardiovascular and cerebrovascular diseases are prevented and treated.

## 1. Introduction

Cardiovascular disease (CVD) and cerebrovascular disease together constitute the leading cause of mortality worldwide [1,2,3]. In 2022 alone, an estimated 19.8 million deaths were attributable to CVD, approximately 32% of all global fatalities, with myocardial infarction and stroke jointly accounting for roughly 85% of that toll [1,3]. The burden falls disproportionately on low- and middle-income countries (LMICs) [1,3,4]. Within this landscape, cardiac arrhythmias, atherosclerotic cardiovascular disease, and ischaemic stroke contribute substantially and are mechanistically intertwined: atrial fibrillation (AF), for instance, markedly amplifies stroke risk and all-cause mortality [5,6], compounding the global burden of both disease categories.

Pharmacotherapy, revascularisation, device-based interventions, and surgery have each advanced outcomes, yet residual risk remains clinically significant, particularly for recurrent stroke and major adverse cardiovascular events, even among patients adherent to guideline-directed therapy [7,8,9]. Anticoagulated patients with AF, for example, retain a non-trivial absolute stroke risk [8,10,11]. More fundamentally, conventional approaches leave core disease drivers intact: pathogenic genetic variants, chronic vascular inflammation, maladaptive cell death, and adverse vascular remodelling continue to sustain atherosclerosis, cardiac dysfunction, and cerebrovascular injury [12,13]. The convergence of an enormous disease burden, persistent residual risk despite optimal therapy, and incompletely addressed causal biology defines a clear therapeutic gap, one that gene-based strategies, by targeting molecular origins, are uniquely positioned to fill.

Gene therapy, broadly encompassing the introduction, silencing, repair, or editing of genetic material to correct disease-causing molecular abnormalities [14,15], has matured from conceptual framework to emerging clinical reality [16]. Early approaches relied principally on adenoviral and lentiviral vectors, which, despite efficient transgene delivery, were constrained by immunogenicity and insertional mutagenesis [16]. The subsequent development of non-viral platforms, particularly lipid nanoparticles and polymer-based carriers, has broadened the delivery repertoire with improved safety and manufacturing scalability [16,17]. In parallel, gene-regulatory modalities, including small interfering RNAs (siRNAs), antisense oligonucleotides (ASOs), and microRNAs (miRNAs), have enabled precise transcriptional and post-transcriptional silencing, with siRNA-based therapeutics now approved for rare metabolic and cardiovascular conditions [18]. Most recently, CRISPR/Cas9, base editors, and prime editors have raised the prospect of permanent, single-administration correction of pathogenic variants [19].

Applied to cardiovascular and cerebrovascular disease, these tools offer a qualitative departure from conventional therapy. Rather than modulating downstream pathophysiology, gene-based interventions act on the causal substrate, whether disordered lipid metabolism, ion channel dysfunction, vascular inflammation, or impaired neuronal survival [20,21], with the potential to modify, rather than merely manage, disease trajectory [16]. This transition from symptomatic palliation toward causal correction, has generated considerable scientific and clinical momentum [16,22].

This review examines the application of gene therapy across three representative and high-burden disease domains: cardiac arrhythmias, atherosclerotic cardiovascular disease, and ischaemic stroke. We first outline the conceptual and technological foundations underpinning current gene-based strategies, then survey their mechanistic rationale and translational evidence within each disease context, and finally identify outstanding challenges and future directions, with the aim of delineating where gene therapy can most meaningfully address unmet needs in cardiovascular and cerebrovascular medicine.

## 2. Cardiac Arrhythmia and Gene Therapy

### 2.1. Background

Cardiac arrhythmia ranks among the most prevalent cardiovascular conditions and represents a growing public health burden [23]. Rhythmic cardiac function depends on the coordinated association of specialised pacemaker and conduction structures; perturbations in either can precipitate arrhythmic disorders [24,25]. Malignant arrhythmias carry significant mortality risk and are the principal substrate of sudden cardiac death (SCD), with profound consequences for both survival and quality of life [24]. Atrial fibrillation (AF) has reached epidemic proportions globally [26,27], ventricular arrhythmias underlie 70–80% of SCD cases worldwide [24], and inherited arrhythmia syndromes are disproportionately concentrated in high-risk populations [28,29].

Standard management encompasses pharmacotherapy, electrical cardioversion, and catheter ablation, each with meaningful limitations [26,27,30]. Class I and III antiarrhythmic agents carry proarrhythmic risk, and long-term amiodarone use, despite its efficacy, incurs thyroid, pulmonary, and hepatic toxicity [31,32]. Implantable devices reduce SCD risk [24] but neither prevent arrhythmia initiation nor spare patients from symptomatic shocks. Catheter ablation offers superior rhythm control in AF [30], yet recurrence is common, and procedural complications, including pericardial tamponade and pulmonary vein stenosis, remain clinically significant concerns [30,33,34]. The shared deficiency of these approaches, their failure to engage the underlying molecular pathology, underscores the need for therapies directed at the genetic and mechanistic roots of malignant arrhythmia.

Arrhythmias constitute a highly heterogeneous disease group, with pathogenesis attributable to ion channel dysfunction, structural protein abnormalities, and disrupted molecular regulatory networks [25,28]. Ion channel gene mutations represent the most frequent genetic aetiology [28]: pathogenic variants in KCNQ1 and KCNH2 cause congenital long QT syndrome (LQTS) [35,36,37], while SCN5A mutations underlie multiple conditions, including long QT syndrome type 3 (LQT3) [38,39,40]. RYR2 mutations are a principal cause of catecholaminergic polymorphic ventricular tachycardia (CPVT), illustrating the central role of calcium-handling genes in arrhythmogenesis [41,42,43,44]. Structural arrhythmias carry an equally significant genetic dimension: mutations in plakophilin-2 (PKP2), desmoplakin (DSP), and desmoglein-2 (DSG2) are the dominant drivers of arrhythmogenic right ventricular cardiomyopathy (ARVC) [45,46,47,48,49], whereas MYH7 and MYBPC3 mutations underlie hypertrophic cardiomyopathy (HCM) [28,29]. Next-generation sequencing has further implicated FLNC and TTN variants, linking structural protein defects to broader arrhythmia susceptibility [28]. Together, these advances in molecular genetics have established a robust conceptual foundation for gene-targeted and precision medicine approaches, and numerous gene therapy trials are currently underway [28,29,50].

### 2.2. Molecular and Genetic Basis for Gene Therapy in Arrhythmias

Despite considerable phenotypic diversity, most inherited and acquired arrhythmias can be organised around four mechanistically interwoven pro-arrhythmic axes. The first is the sodium current–conduction safety margin axis, centred on SCN5A/NaV1.5 with non-coding regulatory input from SCN10A [38,39]. The second is the potassium channel–action potential duration axis, driven by KCNQ1/KCNH2, whose dysfunction prolongs or abbreviates repolarisation and amplifies spatial heterogeneity [35,36,37]. Third is the calcium homeostasis–afterdepolarisation axis, anchored by RYR2-mediated Ca^2+^ release and integrated through CALM1/2/3 signalling, which predisposes to early and delayed afterdepolarisations [42,43,44]. Fourth is the structural–decoupling axis, arising from desmosomal and gap junctional defects, exemplified by PKP2 and TMEM43 mutations in ARVC, that generate conduction discontinuity [45,46,47,51]. These axes converge therapeutically on a shared repertoire of gene-based strategies: transgene replacement, suppression-and-replacement, precision editing, and augmentation of endogenous ionic or bypass currents [25,28,29,50].

#### 2.2.1. The Sodium Current–Conduction Safety Margin Axis

The sodium current (INa) is the primary determinant of cardiac conduction velocity and excitability. SCN5A encodes NaV1.5, which drives the rapid depolarisation phase of the cardiac action potential; loss-of-function variants reduce INa, slow conduction, and impair cell-to-cell excitation, thereby narrowing the conduction safety margin. This predisposes to unidirectional conduction block and re-entry, a fundamental mechanism common to many malignant arrhythmias. SCN5A mutations are causally linked to LQT3, Brugada syndrome, AF, and progressive cardiac conduction disease [40].

SCN10A functions as an important modifier of this axis, though not as a classical depolarising channel. Rather, it operates principally as a non-coding regulatory node via a cardiac-specific enhancer cluster at the SCN5A–SCN10A locus [38]. Although the full-length *SCN10A* transcript is scarce in cardiac tissue, a short isoform (SCN10A-short) is expressed in atrial myocytes, the sinoatrial node, and the cardiac conduction system. Its encoded protein, NaV1.8-short, augments NaV1.5-mediated current, thereby enhancing INa and conduction safety margin [38,39]. Variants disrupting the enhancer reduce SCN10A-short expression, slowing conduction and promoting arrhythmia, without altering SCN5A transcript levels [38]. GWAS signals for AF susceptibility, heart rate, and conduction velocity map to this locus [38], providing molecular grounding for how non-coding variants modulate arrhythmia risk and offering a rational basis for target selection in suppression-replacement and current-augmentation strategies.

Gene therapy on this axis therefore pursues two parallel goals: direct restoration of INa and functional enhancement of the conduction safety margin. AAV9-ABEmax base editing of pathogenic SCN5A variants has demonstrated efficacy in correcting the LQT3 phenotype [40], while SCN10A-short augmentation strategies can restore INa and prevent arrhythmia through a complementary, compensation-based mechanism [38,39]. These converging approaches, precise pathogenic correction alongside functional rescue, illustrate the dual therapeutic logic now emerging in arrhythmia gene therapy.

#### 2.2.2. The Potassium Channel–Action Potential Duration Axis

Cardiac repolarisation is governed principally by potassium currents through channels encoded by KCNQ1 (Kv7.1; IKs) and KCNH2 (Kv11.1; IKr) [35,36,37]. Loss-of-function variants in either gene prolong repolarisation, causing long QT syndrome types 1 and 2 (LQT1/2), whereas gain-of-function mutations abbreviate it, producing short QT syndrome (SQT1) [35,36,37]. Both extremes elevate the risk of early afterdepolarisations (EADs) and repolarisation heterogeneity, creating substrate for re-entry. The suppression-and-replacement (SupRep) strategy addresses this axis by deploying shRNAs to silence mutant alleles while simultaneously delivering an shRNA-resistant replacement cDNA. In iPSC-derived cardiomyocytes and animal models alike, both KCNQ1-SupRep and KCNH2-SupRep normalised action potential duration (APD) to near-physiological levels, establishing potassium channel restoration as a therapeutically tractable target [35,36,37].

#### 2.2.3. The Calcium Homeostasis–Afterdepolarisation Axis

Intracellular calcium dysregulation is a central driver of triggered arrhythmias. RYR2 mutations cause pathological sarcoplasmic reticulum (SR) Ca^2+^ leak, generating delayed afterdepolarisations (DADs) and the characteristic phenotype of catecholaminergic polymorphic ventricular tachycardia (CPVT) [42]. Mutations in CALM1, CALM2, and CALM3 disrupt calmodulin-mediated regulation across multiple ion channels, producing calmodulinopathy that typically manifests as malignant LQTS or CPVT in children and adolescents [42,43]. Gene therapy strategies targeting this axis span several modalities: SupRep combining mutant allele silencing with shRNA-resistant CALM cDNA delivery [43]; ASOs directed against mutant transcripts [44]; and hybrid approaches pairing gene tools with small-molecule Ca^2+^ leak suppressors. Across diverse experimental models, these interventions corrected electrophysiological abnormalities and reduced arrhythmia burden [43,44].

A mechanistically distinct but related vulnerability within this axis involves impaired SR Ca^2+^ reuptake. In heart failure, SERCA2a downregulation reduces SR Ca^2+^ sequestration, elevating diastolic cytosolic Ca^2+^ and promoting spontaneous RyR2-mediated release. The resultant Na^+^/Ca^2+^ exchanger activation generates inward depolarising current, triggering DADs and arrhythmia. AAV-mediated SERCA2a gene transfer counters this cascade by enhancing SR Ca^2+^ reuptake and dampening diastolic Ca^2+^ accumulation, thereby suppressing RyR2 leak and DAD incidence. Contrary to early concerns that increased SR Ca^2+^ loading might be proarrhythmic, preclinical studies have consistently demonstrated reduced ventricular arrhythmia burden across multiple disease models, positioning SERCA2a as an anti-arrhythmic positive inotrope [52]. Collectively, the breadth of effective interventions across RYR2, CALM1–3, and SERCA2a reinforces the calcium homeostasis axis as a high-value therapeutic target.

#### 2.2.4. The Structural–Decoupling Axis

Reliable electrical propagation requires not only functional ion channels but also structural integrity at the intercalated disc. PKP2 and TMEM43 are essential for maintaining both mechanical adhesion and electrical continuity between cardiomyocytes; mutations in these desmosomal and junctional proteins destabilise the intercalated disc, producing conduction discontinuity, fibro-fatty myocardial replacement, and malignant ventricular arrhythmias characteristic of ARVC [45,46,47,51]. Loss of intercellular coupling further synergises with reduced INa to erode the conduction safety margin [45,47,51], collectively favouring unidirectional conduction block and re-entry circuit formation.

AAV-mediated PKP2 replacement restored protein localisation, improved conduction, and prolonged survival in murine models [45,46], while AAV-driven TMEM43 overexpression delayed ARVC5 onset and attenuated fibrosis [51]. Extending the principle of genomic correction to structural cardiomyopathy, in vivo base editing of the PLN-R14del mutation reversed arrhythmia susceptibility in humanised mice [53], demonstrating that structural defects amenable to precise editing can normalise electrophysiological behaviour and suppress re-entry, validating structural proteins as legitimate gene therapy targets.

#### 2.2.5. Therapeutic Convergence Across Axes

##### Direct Causal Correction

The most straightforward gene therapy paradigm is direct repair or replacement of the pathogenic allele, restoring channel function and action potential duration at the source. SupRep exemplifies this logic, achieving APD normalisation across distinct mutation types in KCNQ1- and KCNH2-related repolarisation disorders [35,36,37]. AAV9-ABEmax base editing of SCN5A in LQT3 murine models delivered high-efficiency allele correction, reversing QT prolongation and arrhythmic phenotype [40]. The consistency of these results across mechanistically distinct axes supports the broad feasibility of direct causal correction as a clinical strategy.

##### Current Augmentation and Bypass Compensation

Where direct repair is impractical, strategies that enhance existing currents or introduce compensatory pathways can raise conduction and repolarisation safety margins. On the sodium axis, AAV-delivered SCN10A-short augments NaV1.5-mediated current and restores conduction in animal models [38,39]. Prokaryotic Na^+^ channels, whose compact coding sequences circumvent SCN5A packaging constraints, have shown the capacity to restore action potential conduction and elevate safety margins in both in silico and in vivo settings [54,55,56]. On the calcium axis, SERCA2a gene transfer improves Ca^2+^ handling and suppresses DADs [57], while CALM1–3 SupRep and replacement therapies correct malignant calmodulinopathy phenotypes [43,44]. Upstream regulatory modulation extends this logic further: cardiac PDE2A delivery attenuated adverse remodelling and arrhythmia in heart failure models [58]; shRNA-mediated NOX2 silencing prevented electrical remodelling and AF in a canine model [59]; and adiponectin gene therapy reduced sympathetic overdrive, suppressing ventricular arrhythmias and remodelling post-infarction [60]. In the rare metabolic arrhythmia syndrome TANGO2 deficiency, folate supplementation ameliorated lethal ventricular arrhythmias [61]. Together, these findings establish augmentation and bypass compensation as a versatile strategy spanning sodium, calcium, structural, and metabolic domains.

##### Coupling and Network Restoration

**Figure 1:**
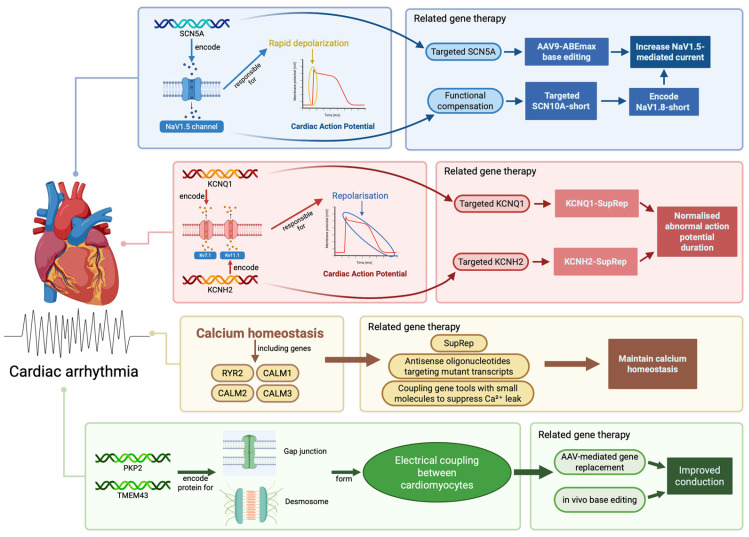
Molecular and genetic basis of cardiac arrhythmias and related gene therapy strategies. Schematic illustration of the four major pro-arrhythmic axes: (i) sodium current–conduction safety margin (SCN5A/NaV1.5; SCN10A-short), (ii) potassium channel–action potential duration (KCNQ1/KCNH2), (iii) calcium homeostasis–afterdepolarisation (RYR2; CALM1–3), and (iv) structural–connective coupling (PKP2; TMEM43). Representative gene therapy modalities, base editing, suppression-and-replacement, antisense oligonucleotides, and gene replacement, are mapped to each axis, highlighting convergent strategies to restore conduction, repolarisation, calcium balance, and structural integrity. (Created in BioRender. Liu, Z. (2026) https://BioRender.com/2qqglu6, accessed on 10 May 2026).

The third therapeutic category targets structural and junctional proteins to re-establish intercellular coupling and tissue-level conduction continuity. PKP2 replacement and TMEM43 overexpression restored intercalated disc integrity, improved conduction, and reduced ventricular arrhythmia burden in murine and cellular ARVC models [45,46,51], while PLN-R14del base editing substantially decreased arrhythmia susceptibility in humanised mice [53]. Connexin-43 (Cx43/GJA1) overexpression further demonstrated that enhancing gap-junctional coupling improves conduction homogeneity and raises the ventricular fibrillation threshold in animal models [62]. Structural restoration, therefore, not only mitigates the direct consequences of uncoupling but also interacts synergistically with current augmentation strategies, establishing it as an indispensable pillar of the arrhythmia gene therapy framework (Figure 1; Table 1).

## 3. Atherosclerosis and Gene Therapy

### 3.1. Background

Atherosclerosis is the pathological substrate of most cardiovascular and cerebrovascular diseases, a chronic, systemic disorder of medium- and large-calibre arteries that remains the world’s leading cause of morbidity and mortality, with myocardial infarction and stroke together accounting for roughly one-third of all deaths, surpassing cancer [17,20,63,64,65]. The disease is initiated by endothelial dysfunction and lipid deposition at arterial sites of disturbed haemodynamic flow, such as bifurcations and curvatures [17,66,67]. This triggers smooth muscle cell proliferation, extracellular matrix remodelling, and immune cell infiltration, culminating in plaque formation [65,68]. Critically, disease progression is driven not only by excess inflammation but also by its defective resolution [69]. As plaques advance, rupture promotes platelet aggregation and thrombosis, precipitating acute events including myocardial infarction, stroke, renovascular hypertension, and peripheral arterial disease [17,63,64,68]. Standard therapies, including statins, antiplatelet and antithrombotic agents, and surgical interventions such as endarterectomy, balloon dilation, and stenting, reduce risk but are limited by side effects and leave core disease mechanisms unaddressed [17], underscoring the need for strategies targeting the molecular and genetic drivers of the disease.

### 3.2. Pathological Mechanisms and Therapeutic Targets

Lipid metabolism occupies the central pathogenic role in atherosclerosis, with the LDLR pathway and PCSK9 as its principal regulators. The disease is fundamentally driven by retention of apolipoprotein B, the main structural component of LDL-C, within the arterial intima [70], a process governed by hepatic LDLR. Under physiological conditions, LDLR binds ApoB-100 on circulating LDL particles, mediates endocytosis, and recycles to the hepatocyte surface for repeated uptake [71]. This clearance efficiency is critically modulated by PCSK9, which binds the EGF-A domain of LDLR and diverts the receptor to lysosomal degradation rather than recycling [72]. When LDLR function is impaired, as in familial hypercholesterolaemia, plasma LDL-C rises markedly, accelerating intimal LDL deposition and atherogenesis [70].

Inflammatory signalling constitutes a second major pathogenic axis. NF-κB activation drives transcription of IL-1β and MCP-1, amplifying vascular inflammation, promoting monocyte recruitment, and accelerating foam cell formation and lesion growth [73,74,75,76]. The CANTOS trial provided direct clinical validation of this pathway by demonstrating that IL-1β inhibition with canakinumab significantly reduced recurrent cardiovascular events independently of lipid lowering [73,77]. Endothelial dysfunction represents a third axis, intimately linked to impaired eNOS signalling and dysregulated VEGF activity. Members of the VEGF family, including VEGF-A, VEGF-C, and VEGF-D, influence angiogenesis, lymphangiogenesis, oxidative stress, inflammation, and lipid metabolism throughout atherosclerotic progression [78,79]. VEGF-A in particular increases endothelial permeability through both eNOS-dependent and eNOS-independent mechanisms [80], while reduced nitric oxide bioavailability, a downstream consequence of eNOS impairment, substantially contributes to endothelial dysfunction in hypertension, diabetes, and hyperlipidaemia [81].

Hereditary susceptibility constitutes a fourth axis of increasing relevance. Large cohort analyses using imaging-defined phenotypes have uncovered three novel genomic regions and 20 independent loci, including established genes such as *CDKN2B-AS1*, *APOE*, *LPA-PLG*, *PHACTR1*, and *COL4A1-COL4A2* [82,83]. Over 300 risk loci for coronary artery disease have now been mapped, delineating the polygenic architecture of cardiovascular risk [82]. Transcriptome-wide association studies (TWAS) have further linked genetic variants to functional expression profiles relevant to atherosclerosis [84], while meta-GWAS analyses have replicated approximately 150 established loci and identified 54 novel ones, deepening insight into disease pathobiology [85].

### 3.3. Gene Therapy Targeting Lipid Metabolism

Most gene therapy advances in atherosclerosis to date have concentrated on lipid metabolism, particularly the LDLR–PCSK9 axis. Preclinical studies have demonstrated that AAV-mediated *LDLR* augmentation achieves profound and durable LDL-C reduction: in double-knockout mouse models, codon-optimised, degradation-resistant LDLR constructs delivered via AAV8 lowered plasma LDL-C by nearly 98% at relatively low doses, with stable expression maintained for at least 120 days [86]. AAV8-mediated LDLR delivery also showed significant efficacy in homozygous mice carrying the human HoFH pathogenic mutation Ldlr p.W483X [30], and the consistent efficacy and safety of AAV8.TBG.hLDLR has been further confirmed in humanised HoFH models [87]. Notably, AAV-LDLR efficacy in LDLR^−^/^−^ mice could be further enhanced by combination with AAV-PCSK9 inhibition [88], suggesting a rationale for dual-targeting approaches.

Among gene-silencing strategies, inclisiran, a GalNAc-conjugated siRNA targeting hepatic *PCSK9* mRNA, is currently the most clinically advanced, reducing PCSK9 synthesis and thereby facilitating LDLR recycling and LDL-C clearance [18,89,90,91]. The ORION clinical programme has systematically established its efficacy: the phase 2 ORION-1 trial demonstrated durable LDL-C reductions with infrequent dosing over 6–12 months [92]; ORION-9 confirmed significant LDL-C lowering versus placebo in adults with heterozygous familial hypercholesterolaemia (HeFH) [89]; and the pivotal phase 3 trials ORION-10 and ORION-11 achieved placebo-adjusted LDL-C reductions of approximately 52% and 50%, respectively, at day 510 under a day-1/day-90/every-6-month schedule [18]. The open-label ORION-3 extension confirmed that twice-yearly dosing sustains LDL-C and PCSK9 reduction over four years [90], and the ORION-8 extension, encompassing more than 12,000 patient-years of exposure, demonstrated maintained efficacy with a favourable long-term safety profile at population scale [91]. Across these trials, inclisiran consistently achieves approximately 50% sustained LDL-C reduction, establishing it as the first-in-class gene-silencing therapy for hypercholesterolaemia, though injection-site reactions and the absence of long-term cardiovascular outcome data remain important caveats.

For one-time permanent inhibition, PCSK9 has been the first target subjected to rigorous gene-editing validation. Early CRISPR–Cas9 knockout studies in animal models confirmed both feasibility and substantial LDL-C reduction [93]. Building on this, Musunuru and colleagues advanced to non-human primates in 2021, demonstrating that LNP-delivered ABE8.8 with a PCSK9 guide RNA produced robust, durable reductions in circulating PCSK9 and LDL-C, with comprehensive off-target analyses confirming high editing specificity [94]. This work underpinned the clinical candidate VERVE-101, LNP-encapsulated ABE8.8 mRNA paired with a PCSK9 guide RNA, which achieved dose-dependent, sustained LDL-C reduction with a favourable preclinical safety profile [95]. In the phase 1b HEART-1 trial, a single VERVE-101 infusion produced dose-dependent reductions in PCSK9 (up to ~84%) and LDL-C (up to ~55%) in patients with HeFH; however, transient liver transaminase elevations and treatment-related adverse events were reported, underscoring the translational challenges of delivery specificity, immunogenicity, and long-term safety that remain to be resolved [96].

Distinct from permanent base substitution, epigenetic editors represent an emerging complementary approach. The evolved engineered transcriptional repressor (EvoETR) silences PCSK9 via targeted DNA methylation without sequence alteration, producing durable LDL-C lowering in murine and non-human primate models that is reversible upon demethylation, a unique advantage of tunable, on/off control [19,97]. In parallel, ASOs against *PCSK9*, notably AZD8233, have achieved LDL-C reductions of 70–80% in phase 2b trials [98,99], further illustrating the complementary potential of diverse RNA-based modalities.

Collectively, these advances trace a coherent therapeutic trajectory from transient gene silencing through permanent editing to reversible epigenetic modulation, positioning PCSK9 as a paradigm-setting target in cardiovascular gene therapy, while acknowledging that long-term efficacy, safety, and clinical scalability remain to be fully established.

### 3.4. Gene Therapy Targeting Inflammatory Pathways

**Figure 2:**
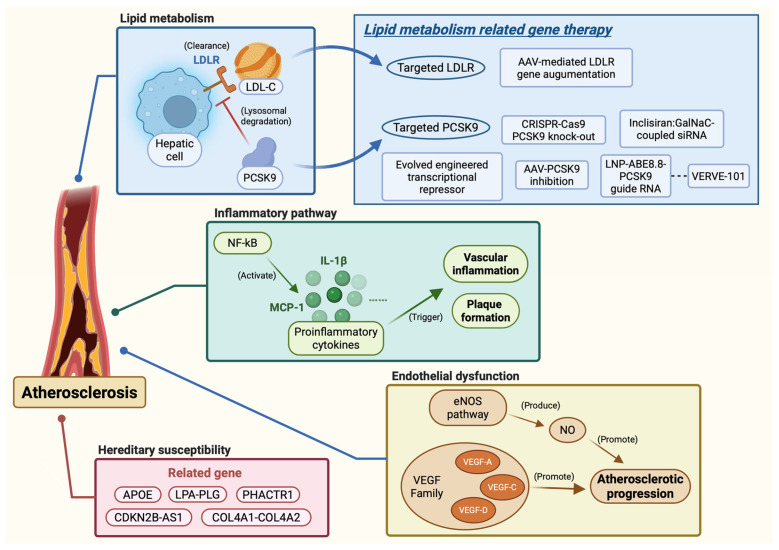
Pathological mechanisms and gene therapy strategies in atherosclerosis. The disease arises from four major axes: lipid metabolism (LDLR-mediated LDL-C clearance impaired by PCSK9), inflammation (NF-κB activation driving IL-1β and MCP-1 release), endothelial dysfunction (impaired eNOS/NO signalling and dysregulated VEGF activity), and hereditary susceptibility (risk loci including APOE, LPA–PLG, PHACTR1, CDKN2B-AS1, and COL4A1–COL4A2). Current gene therapy strategies principally target lipid metabolism, encompassing AAV-mediated LDLR augmentation, PCSK9 silencing by siRNA (inclisiran), CRISPR–Cas9 knockout, base editing (ABE8.8; VERVE-101), and epigenetic repression. (Created in BioRender. Liu, Z. (2026) https://BioRender.com/gw9pow0, accessed on 10 May 2026).

Beyond lipid metabolism, inflammatory pathways constitute a biologically and clinically validated therapeutic axis in atherosclerosis. NF-κB-driven transcription of IL-1β and MCP-1 sustains vascular inflammation, accelerates monocyte recruitment, and promotes foam cell formation and plaque instability [73,74,75,76,77]; IL-1β, as a central downstream effector, plays a particularly prominent role in propagating inflammatory signalling throughout lesion progression [73]. The CANTOS trial’s demonstration that canakinumab-mediated IL-1β inhibition reduced recurrent cardiovascular events independently of lipid lowering provides direct clinical evidence that targeting this axis yields meaningful benefit [77]. Despite this validation, gene therapy approaches directed specifically at vascular inflammatory pathways remain at an earlier stage of development than lipid-targeting strategies, and their clinical translation is still limited. Further work is needed to establish the feasibility, target specificity, and safety of gene-based inflammatory modulation in the atherosclerotic vessel wall (Figure 2; Table 2).

## 4. Ischaemic Stroke and Gene Therapy

### 4.1. Background

Ischaemic stroke accounts for approximately 62–70% of all stroke cases and represents a major and growing global health burden [100]. Data from the 2021 and 2024 Global Burden of Disease studies and the World Stroke Organisation collectively document substantial increases in stroke incidence, mortality, and disability-adjusted life years between 1990 and 2021 [4,101]. Current standard-of-care interventions, intravenous thrombolysis (IVT) and mechanical thrombectomy (MT) [102,103,104], are constrained by narrow therapeutic time windows [105,106], and effective reperfusion remains inaccessible to the majority of patients in low- and middle-income countries [107].

The pathological sequelae of ischaemic stroke span acute-phase events, neuroinflammation, cell death, and blood–brain barrier (BBB) disruption, and chronic limitations in neuroregeneration, together driving persistent functional deficits [104,108,109,110]. No current pharmacological therapy adequately addresses this full mechanistic spectrum. Gene-based interventions, by modulating disease-relevant pathways at the molecular level, offer the prospect of overcoming these inherent limitations of conventional treatment [111].

### 4.2. Molecular Pathophysiology Relevant to Gene Therapy

Two major pathological axes underlie the injury and recovery trajectory following ischaemic stroke, each providing well-defined entry points for gene-based intervention.

Neuroinflammation is a dominant feature of the acute phase. The cGAS–STING pathway is activated by cytosolic DNA released from ischaemia-damaged mitochondria and nuclei; downstream TBK1 and IRF3 phosphorylation drives type I interferon production and NF-κB-mediated inflammatory amplification, promoting microglial activation and oligodendrocyte injury that collectively worsen white matter damage [112]. Inhibiting this pathway attenuates inflammation, facilitates remyelination, restores white matter integrity, and improves motor recovery across multiple experimental models [113,114,115,116]. LCP1, an emerging inflammatory regulator, is highly expressed in monocyte-derived macrophages (MoDMs) after stroke; single-cell transcriptomic and CyTOF analyses show that its knockdown reduces neuroinflammatory infiltration, attenuates systemic immune suppression, and remodels AKT/EGFR and lipid–glucose metabolic signalling [117,118]. Pro-inflammatory cytokines IL-1β and TNF-α are also markedly elevated post-stroke, with sustained upregulation linked to multi-organ complications and poor prognosis [119,120].

Impaired neuroregeneration constitutes the second major axis. Excessive astrocyte proliferation and migration produce glial scarring with attendant neurotoxic factor release and axonal growth inhibition [121], while disrupted oligodendrocyte progenitor differentiation and increased phagocytic cell burden limit remyelination and white matter repair [114,122]. cGAS–STING inhibition has been shown to restore oligodendrocyte progenitor differentiation and improve long-term structural outcomes [114]. Reduced expression of neurotrophic factors, particularly VEGF and BDNF, further restricts neural stem cell proliferation and differentiation [104]. VEGF promotes angiogenesis and tissue repair, while BDNF activates TrkB signalling to support neuronal survival, synaptic plasticity, and axonal regeneration; elevated circulating VEGF in acute stroke models has been associated with improved neural recovery, affirming its therapeutic relevance [123].

### 4.3. Therapy Strategies for Ischaemic Stroke

#### 4.3.1. Targeting Inflammatory Pathways

Pathological cGAS–STING activation is a principal driver of post-ischaemic neuroinflammation and tissue injury, and genetic silencing of this axis has demonstrated consistent neuroprotective efficacy across mechanistically distinct stroke models. In adult MCAO/reperfusion mice, lentiviral cGAS-siRNA delivery markedly suppressed inflammatory cascades, curtailed pro-inflammatory cytokine release, and reduced infarct volume with improved neurological outcomes [124]. STING-siRNA administration in neonatal hypoxic–ischaemic encephalopathy models similarly alleviated neuronal necrosis and enhanced long-term functional recovery [125], while intranasal or intraventricular STING-siRNA in a murine cerebral venous sinus thrombosis (CVST) model attenuated neuroinflammation and improved outcomes across multiple endpoints [126]. LCP1 silencing in MoDMs via siRNA or shRNA further reduced infarct volume and improved both motor and cognitive performance in mice [118], extending the genetic silencing paradigm to a distinct inflammatory mediator. Collectively, these findings establish innate immune gene silencing as a neuroprotective strategy applicable across stroke subtypes and injury phases.

Cytokine-level modulation has been pursued through complementary upregulation of anti-inflammatory mediators and suppression of pro-inflammatory effectors. IL-10 acts primarily through JAK1–STAT3 activation to suppress pro-inflammatory cytokine production and limit microglial activity, reducing secondary neuronal injury [127]. VCAM-1-targeted LNPs carrying IL-10 mRNA achieved selective expression in activated vascular endothelium and immune cells after stroke, reducing infarct size and improving survival [128]. Earlier work showed that intracerebroventricular Ad-IL-10 elevated CSF IL-10 concentrations and attenuated infarct and hippocampal damage in rodents, and AAV-IL-10-modified mesenchymal stem cells conferred further neuroprotection in animal models [129,130]. On the suppressive side, AAV-shRNA targeting C/EBPβ and its downstream effectors IL-1β and TNF-α effectively reduced inflammation-driven neurotoxicity and tissue injury in murine ischaemic models [131].

EV-mediated miRNA delivery has rapidly emerged as a third and mechanistically distinct anti-inflammatory strategy, with miR-124 the most extensively characterised cargo in stroke models. miR-124 drives microglial polarisation toward an anti-inflammatory phenotype, suppresses NF-κB, and modulates STAT3 and NOTCH signalling to facilitate neurogenesis and astrocyte reprogramming [132]; circulating and CSF miR-124 levels additionally show promise as prognostic biomarkers of injury severity and functional recovery [133]. M2 microglia-derived EVs carrying miR-124 reduced infarct volume and suppressed inflammation through USP14 targeting [134], while M2-derived small EVs enriched in miR-124 inhibited astrocyte proliferation via the STAT3 pathway, promoted reprogramming into neural progenitor-like cells, curtailed glial scarring, and enhanced long-term tissue repair [135]. More recently, miR-124-loaded EVs were shown to drive endogenous neurogenesis by promoting NSC proliferation and differentiation into mature neurons through the AAK1–NOTCH pathway, facilitating functional reconstruction [136]. The inherent BBB-crossing capacity, biocompatibility, and immune privilege of EVs position them as particularly well-suited vectors for CNS gene therapy in stroke. From innate immune silencing and cytokine modulation to EV-based miRNA delivery, these complementary approaches furnish converging preclinical evidence that neuroinflammation is among the most actionable therapeutic axes for gene therapy in ischaemic stroke.

#### 4.3.2. Neuroregeneration and Repair

Transcription factor-driven in vivo reprogramming has become a leading strategy for structural post-stroke repair, with AAV-NeuroD1 accumulating the broadest and most consistent preclinical validation. NeuroD1 is a bHLH transcription factor central to neural stem and progenitor cell differentiation into mature neurons [137]. In non-human primate MCAO models, AAV9-GFAP::NeuroD1 delivered via a Cre-FLEX system successfully reprogrammed reactive astrocytes into neurons and protected parvalbumin-positive interneurons within the conversion zone; crucially, treatment initiated 10–30 days post-stroke still promoted new neuron survival and cortical structural recovery [138]. In rats with chronic large-volume stroke, repeated AAV-NeuroD1 injections combined with complementary transcription factors regenerated neurons across grey and white matter, reduced glial scarring, restored white matter integrity, and produced sustained behavioural improvements [139]. Extending the reprogramming substrate beyond glia, AAV-ShH19-NeuroD1 converted microglia and macrophages within the ischaemic core into GABAergic neurons, accompanied by microenvironmental improvement and significant motor recovery [140]. Across these models, AAV-NeuroD1 consistently demonstrates the capacity to circumvent the acute therapeutic window and deliver meaningful neural repair through the subacute and chronic post-stroke phases.

Epigenetic modulation offers a complementary repair strategy that avoids permanent sequence alteration. Intranasal delivery of a CRISPR-dCas9-VP64 transcriptional activation system encapsulated in CaP/PEI-PEG-bHb nanoparticles upregulated endogenous Sirt1 in pMCAO mice, markedly reducing cerebral oedema, attenuating neuroinflammation, and improving survival [141]. Sirt1 confers neuroprotection by deacetylating NF-κB and supporting mitochondrial function, thereby reducing apoptosis and enhancing cellular resilience [142]. Because this approach amplifies endogenous gene activity rather than editing the genome, it affords tunable efficacy with a favourable safety profile; the minimally invasive intranasal route further enhances its practical appeal for acute-phase intervention.

**Figure 3:**
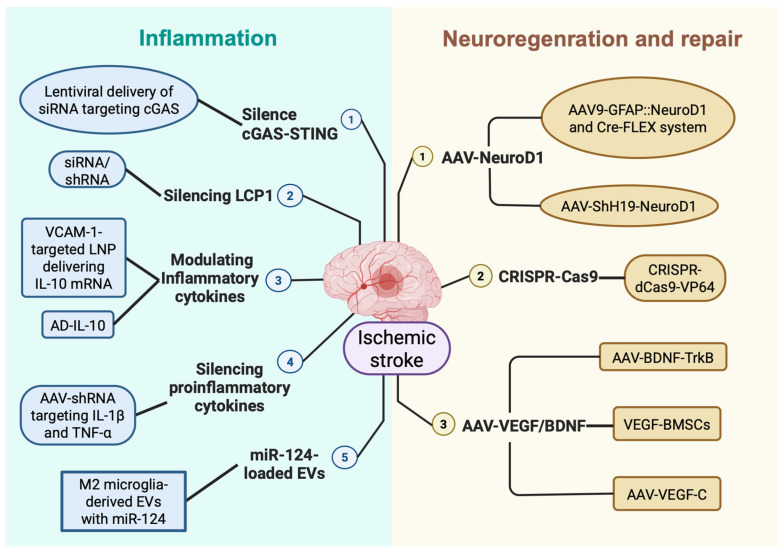
Gene therapy strategies in ischaemic stroke. Approaches are organised into two principal categories, inflammation control and neuroregeneration/repair, encompassing cGAS–STING and LCP1 silencing, IL-10 upregulation, pro-inflammatory cytokine suppression, and EV-delivered miR-124, alongside AAV-NeuroD1-mediated glial reprogramming, CRISPR-dCas9-VP64 epigenetic Sirt1 activation, and VEGF/BDNF gene therapies supporting vascular remodelling and neuronal recovery. (Created in BioRender. Liu, Z. (2026) https://BioRender.com/7w1zark, accessed on 10 May 2026).

Neurotrophic factor gene delivery constitutes the third pillar of neuroregeneration-focused gene therapy. Combined intramuscular AAV-BDNF and intranasal AAV-TrkB delivery strengthened corticospinal tract synaptic connectivity and conduction efficiency in pMCAO rats, producing marked forelimb motor and synaptic transmission recovery [143]. Transplantation of VEGF-modified bone marrow-derived mesenchymal stem cells (VEGF-BMSCs) reduced infarct volume, elevated BDNF levels, improved motor outcomes, attenuated BBB disruption, promoted angiogenesis within the ischaemic penumbra, and decreased neuronal degeneration in peri-infarct regions compared with unmodified BMSCs [144]. Intracerebroventricular AAV-VEGF-C administered as pre-treatment enhanced meningeal lymphatic drainage and upregulated BDNF signalling, mitigating microglial inflammation and improving motor outcomes after stroke [145]. VEGF- and BDNF-based gene therapies thus operate across multiple recovery dimensions simultaneously, acute neuroprotection, vascular remodelling, and sustained neuroregeneration, making them valuable components of a combinatorial gene therapy approach for stroke. Together, neuronal reprogramming, epigenetic activation, and neurotrophic factor delivery collectively expand the therapeutic reach of gene-based interventions well beyond what reperfusion alone can achieve (Figure 3; Table 3).

## 5. Other Cardiovascular and Cerebrovascular Diseases

Beyond the three principal disease domains discussed above, gene therapy is being investigated across a wider spectrum of cardiovascular and cerebrovascular conditions. In heart failure, AAV-mediated transfer of calcium-handling genes, most notably SERCA2a, has been extensively studied, with early clinical data suggesting functional cardiac improvements, though long-term efficacy remains to be established [146,147]. Genetic cardiomyopathies, including hypertrophic and dilated forms, represent compelling targets for editing and replacement strategies aimed at correcting mutations in sarcomeric and structural proteins [148]. Gene-based modulation of vascular tone and renin–angiotensin signalling has been explored in hypertension, though these approaches remain largely preclinical [149]. In cerebrovascular disease beyond ischaemic stroke, several disease-relevant molecular pathways continue to be evaluated as candidate targets for future gene-based intervention [150].

## 6. Shared Mechanisms and Cross-Disease Therapeutic Strategies

### 6.1. Overlapping Pathological Axes and Therapeutic Convergence

Despite marked differences in clinical presentation and tissue-specific pathology, cardiac arrhythmias, atherosclerosis, and ischaemic stroke share several mechanistic threads that carry direct therapeutic implications. Inflammation is the most pervasive. In atherosclerosis, inflammatory signalling drives endothelial activation, immune cell recruitment, and plaque progression; in ischaemic stroke, neuroinflammation mediates secondary neuronal injury, BBB disruption, and impaired tissue repair [68,73,108,120,127,145]; and in arrhythmogenic heart disease, inflammatory and oxidative stress pathways exacerbate electrical remodelling and structural substrate formation [59]. This commonality suggests that gene-based interventions targeting upstream inflammatory nodes may carry therapeutic relevance across multiple disease contexts rather than being purely disease-specific.

Endothelial dysfunction and vascular injury constitute a second mechanistic bridge, linking atherosclerosis and cerebrovascular disease. Impaired endothelial homeostasis promotes lipid retention, vascular inflammation, and plaque destabilisation in the former, while neurovascular unit disruption after stroke worsens cerebral perfusion, BBB integrity, and post-ischaemic recovery in the latter [20,67,108,109]. Strategies that restore vascular function, modulate inflammatory signalling, or support neurovascular repair thus have potential relevance beyond any single disease.

At the therapeutic level, gene replacement and augmentation, RNA interference and antisense-mediated transcript suppression, and genome or epigenome editing represent a shared methodological repertoire applied across all three conditions [15,16,22]. Clinical maturity, however, varies considerably: lipid-lowering approaches targeting PCSK9 and LDLR are the most advanced, with approved and late-stage agents [18,86,89,90,91], whereas gene-based modulation of inflammatory, endothelial, and neurorepair pathways remains predominantly preclinical. This disparity reflects both the promise and the current ceiling of the field.

### 6.2. Delivery Systems

Delivery is not a secondary consideration in gene therapy; it is a primary determinant of efficacy. Therapeutic nucleic acids and gene-editing machinery are biologically inert unless efficiently transported to the appropriate target cells, and the cardiovascular and cerebrovascular systems present particularly demanding delivery environments: the heart, vascular wall, liver, and CNS differ substantially in tissue accessibility, cellular composition, and biological barriers. An effective delivery platform must therefore protect genetic cargo from degradation, achieve meaningful tissue selectivity, minimise immune activation, and sustain gene modulation for a duration appropriate to the therapeutic goal [151].

Gene delivery platforms divide broadly into viral and non-viral systems, each with distinct mechanistic advantages and practical trade-offs. Among viral vectors, encompassing AAV, lentivirus, and adenovirus, AAV has become the dominant platform in cardiovascular and neurological gene therapy, valued for its durable transgene expression, broad tissue tropism, and comparatively favourable safety profile. It has been applied across arrhythmogenic cardiomyopathy, heart failure, and experimental stroke models to restore or supplement disease-relevant genes. Its principal liabilities, pre-existing neutralising antibodies, capsid immunogenicity, a packaging capacity ceiling of approximately 4.7 kb, and incomplete tissue selectivity, continue to constrain clinical translation and represent active areas of vector engineering [15,16]. Non-viral platforms, including lipid nanoparticles, polymer-based carriers, inorganic nanoparticles, and extracellular vesicles, offer a complementary profile: lower immunogenicity, transient expression kinetics that reduce the risk of prolonged vector persistence, and manufacturing scalability amenable to clinical production [22,128,152]. These properties make them particularly well-suited to siRNA, mRNA, ASO, and gene-editing payloads, where durable genomic integration is neither necessary nor desirable.

The application of each platform class is most instructive when considered against specific disease contexts. In hepatic lipid-lowering gene therapy for atherosclerosis, LNP-encapsulated siRNA and base-editing constructs targeting PCSK9 illustrate how non-viral delivery can be tailored to organ-accessible targets with high efficiency and a transient safety profile. In the CNS, the BBB imposes a substantially higher delivery barrier; targeted nanoparticles and EV-based systems are consequently being developed with surface modifications designed to facilitate transcytosis and enable delivery of anti-inflammatory or neurorepair cargo to ischaemic brain tissue. For cardiac indications requiring long-term structural correction, as in channelopathies or cardiomyopathies, AAV retains a practical advantage through persistent episomal expression without the insertional mutagenesis risk of integrating vectors.

Delivery system selection should therefore be governed by three intersecting considerations: the target tissue and its biological accessibility, the therapeutic modality and its expression duration requirements, and the clinical scalability of the manufacturing process. Long-term causal correction of inherited defects favours viral platforms capable of stable, durable expression; transient modulation of inflammatory, metabolic, or repair pathways is better matched to RNA-based or nanoparticle-mediated approaches. Ultimately, advances in cardiovascular and cerebrovascular gene therapy will depend as much on engineering safer, more tissue-selective, and clinically scalable delivery systems as on the identification of new molecular targets, the two challenges are inseparable.

## 7. Challenges and Future Directions

### 7.1. Clinical Significance

Gene-based interventions are fundamentally reorienting therapeutic thinking in cardiovascular and cerebrovascular medicine, shifting the ambition from symptomatic control toward causal, disease-modifying correction. Conventional pharmacotherapy, devices, and revascularisation frequently leave substantial residual risk unaddressed, most strikingly exemplified by recurrent ischaemic stroke in anticoagulated AF patients [10]. Gene therapies, by durably modulating upstream molecular drivers, offer a qualitatively different approach to closing this gap.

In lipid-driven atherosclerosis, this principle has already reached clinical practice: PCSK9-targeting RNA interference delivers sustained LDL-C reduction with infrequent dosing [153], while next-generation in vivo editing and epigenetic silencing aim at one-time or programmable causal target control [19,154]. These advances position gene therapy not as a replacement for guideline-directed care, but as a complementary layer capable of addressing the pathobiology that standard therapy leaves intact. In cardiac arrhythmias, mechanistic organisation into four pro-arrhythmic axes, sodium-conduction, potassium-repolarisation, calcium-triggered activity, and structural coupling, enables target-to-therapy alignment with a precision that pharmacology rarely achieves. SupRep normalises repolarisation by simultaneously silencing mutant alleles and restoring shRNA-immune cDNA, while SCN5A base editing rescues the LQT3 phenotype, together illustrating how precise correction and functional compensation can converge toward durable rhythm control [35,36,37,40]. These strategies also foreshadow genotype-stratified patient selection and combinatorial designs, pairing current augmentation with structural substrate repair, to address heterogeneous arrhythmic risk more comprehensively.

In ischaemic stroke, gene therapy extends the therapeutic reach well beyond the reperfusion window by targeting secondary injury and long-term repair. AAV-NeuroD1-mediated reprogramming of reactive glia into neurons, validated in non-human primate models, alongside gene delivery of BDNF/TrkB and VEGF to support vascular and white matter recovery demonstrate that meaningful functional gains remain achievable after the acute phase has passed [138,145,155]. Vector- or RNA-mediated silencing of innate immune sensors such as cGAS–STING, combined with cytokine modulation, provides multi-layered neuroinflammation control that strongly influences outcome [156]. Collectively, across arrhythmia, atherosclerosis, and stroke, gene therapy offers three distinct but complementary contributions: reducing lifetime event risk through editing or silencing of validated causal nodes such as PCSK9 [19,153,154]; re-establishing electrophysiological safety margins via channel correction or coupling substrate repair [35,36,37,40]; and reopening recovery windows in the injured brain by coupling anti-inflammatory control with cell-fate reprogramming [138,145,155,156]. The clinical significance lies not merely in incremental efficacy gains but in the potential to redesign treatment pathways, from episodic pharmacotherapy toward durable, potentially once-only interventions integrated with precision diagnostics and secondary prevention.

### 7.2. Limitations and Challenges

Despite this progress, substantial barriers to clinical translation remain. Delivery efficiency and safety represent the most immediate constraint. Current vectors, AAV and LNPs, exhibit limited myocardial transduction efficiency and restricted BBB penetrance, and uneven intratissue distribution compromises the spatial consistency of gene expression needed for reliable therapeutic effect [15,157]. Long-term safety remains an open question: immune responses to viral capsids, insertional mutagenesis risk, and off-target editing events, particularly relevant for CRISPR–Cas systems, have not been adequately characterised beyond short follow-up windows, and the absence of extended longitudinal data limits confidence in both durability and safety [158].

Disease-specific complexity adds further layers of difficulty. Arrhythmias involve multiple genes and intersecting pathological axes, making single-gene interventions insufficient for many phenotypes. Atherosclerosis requires coordinated modulation of lipid metabolism, inflammation, and genetic predisposition, suggesting that combinatorial or sequentially targeted approaches will be necessary. In ischaemic stroke, the divergent treatment requirements of the acute injury phase and the chronic repair phase, combined with the persistent challenge of CNS delivery, impose constraints that no current platform fully resolves [15,156,157]. Clinical translation barriers extend beyond biology. Most gene therapy strategies reviewed here, including PCSK9 siRNA (inclisiran), ASO therapies, SupRep, and AAV-NeuroD1, remain in early-phase trials or preclinical development, and large multi-centre validation is still lacking [138,153]. Unresolved regulatory, safety, and patient-selection questions perpetuate this gap. Specific concerns include hepatotoxicity, unintended gene silencing, vector or LNP immunogenicity, and the risks inherent to prolonged or irreversible gene expression. Genetic polymorphisms and phenotypic heterogeneity further reduce the generalisability of single-target strategies, AF illustrates this clearly: as a complex, multifactorial arrhythmia driven predominantly by acquired substrate remodelling rather than monogenic defect, it resists conventional gene therapy frameworks, though modulation of individual genetic susceptibility factors may still offer meaningful risk reduction. Finally, ethical and societal considerations, encompassing the irreversibility of genomic editing, access inequity particularly in LMICs, and the need for robust informed consent and post-market surveillance frameworks, add further complexity to implementation at scale [158].

### 7.3. Future Directions

Realising the clinical potential of cardiovascular and cerebrovascular gene therapy will require parallel and mutually reinforcing advances across technology, clinical evidence generation, and precision medicine integration. On the technological front, myocardium-targeted nanoparticles, engineered AAV capsids with enhanced tissue selectivity, and reversible epigenetic editors are anticipated to improve specificity, broaden the therapeutic index, and reduce off-target risk [15,19]. Clinically, the field urgently needs large, multi-centre, randomised controlled trials; the current evidence base, dominated by small or early-phase studies, is insufficient to support broader adoption or regulatory approval, and only robust long-term trial data can bridge that gap [153].

Integration with precision medicine frameworks will be equally important. Genomic sequencing, multi-omics profiling, and advanced imaging can guide patient stratification and identify those most likely to benefit, particularly in polygenic conditions such as atherosclerosis. Single-cell RNA sequencing and spatial transcriptomics offer particular promise here: single-cell approaches resolve disease-relevant cellular heterogeneity, identify rare pathogenic populations, and define cell-type-specific molecular targets, while spatial transcriptomics preserves tissue architecture and maps where these targetable cell states reside within plaques, infarcted myocardium, or injured brain tissue [159,160,161]. Such personalised approaches will enhance efficacy while avoiding unnecessary risks [153]. Integrating these data modalities can sharpen patient stratification, refine target selection, and guide delivery optimisation across cardiovascular and cerebrovascular applications. Cross-disease strategies exploiting shared pathological mechanisms, most notably the inflammatory axis, may enable a single intervention to confer benefit across multiple conditions, improving the cost–benefit calculus of development. Ultimately, the most transformative shift would be extending gene therapy’s role from acute treatment into prevention and rehabilitation, a transition that would fundamentally reshape long-term care pathways for both cardiac and neurological disease, and that depends on precisely the combination of technological innovation, rigorous clinical validation, and precision patient selection outlined above.

## Figures and Tables

**Table 1:** Summary of gene therapy approaches for cardiac arrhythmia.

Gene/Pathway Target	Modality	Mechanism/Therapeutic Goal	Target Endpoints and Diseases	Status	Citation
*SCN5A*	Base editing (AAV9-ABE)	Correct pathogenic variant to restore INa and repolarisation	Long QT syndrome type 3 (LQT3)	Preclinical	[40]
*SCN10A*	Functional augmentation strategy (vector-mediated delivery of SCN10A-short)	Boost NaV1.5-mediated sodium current	Prevent arrhythmia	Preclinical	[38]
*KCNQ1/KCNH2*	Suppression-and-Replacement (SupRep) via shRNA + replacement cDNA	Silence mutant allele(s) and replace with shRNA-immune WT to normalise APD	Long QT syndrome type 1/2 (LQT1/2), Short QT syndrome (SQT1)	Preclinical	[35,36,37]
*CALM1/CALM2/CALM3*	(i) SupRep (shRNA + replacement cDNA); (ii) ASOs	Restore calmodulin regulation, correct repolarisation defects	Malignant LQTS, CPVT (children/adolescents)	Preclinical	[43]
*PKP2*	AAV-mediated gene replacement	Restore desmosomal protein localisation and conduction coupling	ARVC (desmosomal cardiomyopathy)	Preclinical	[45,46]
*TMEM43*	AAV overexpression (WT)	Augment TMEM43 to delay disease onset and reduce fibrosis	ARVC type 5	Preclinical	[51]
*PLN*	In vivo base editing	Correct pathogenic PLN-R14del mutation at genomic level	Structural cardiomyopathy associated with PLN mutations	Preclinical	[53]
*NOX2*	AAV-shRNA (targeted)	Suppress oxidative injury to prevent electrical remodelling/onset of AF	Atrial fibrillation (AF)—upstream modulation	Preclinical	[59]

**Table 2:** Summary of gene therapy approaches for atherosclerosis.

Gene/Pathway Target	Modality	Mechanism/Therapeutic Goal	Target Endpoints and Diseases	Status	Citation
*LDLR*	AAV-mediated gene augmentation (codon-optimised, degradation-resistant)	Restore LDL receptor function, enhance LDL clearance, reduce plasma LDL-C	HoFH/severe dyslipidaemia	Preclinical	[86]
*PCSK9*	siRNA (GalNAc-conjugated, liver-targeted)	Silence hepatic PCSK9 mRNA to increase LDLR recycling	Hypercholesterolaemia, ASCVD, HeFH	Approved (multiple regions)	[18]
	CRISPR–Cas9 nuclease editing	Knockout PCSK9 to permanently lower LDL-C	Atherosclerosis, hypercholesterolaemia	Preclinical	[93]
	Base editing (ABE8.8, LNP delivery; therapeutic candidate VERVE-101 = LNP-ABE8.8 mRNA + gRNA)	Permanent PCSK9 loss-of-function via base substitution, and LDL-C reduction	Atherosclerosis, hypercholesterolaemia	Preclinical/translational	[94,95]
	Epigenetic editing (DNA methylation, EvoETR)	Reversible silencing of PCSK9 via targeted methylation (on–off control, without DNA alteration)	Atherosclerosis, hypercholesterolaemia	Preclinical	[19,97]
	Antisense oligonucleotide	Target PCSK9 mRNA to suppress protein expression, reduce LDL-C	Hypercholesterolaemia	Phase 2b	[98,99]

**Table 3:** Summary of gene therapy approaches for ischaemic stroke.

Gene/Pathway Target	Modality	Mechanism/Therapeutic Goal	Target Endpoints and Diseases	Status	Citation
cGAS-STING pathway	siRNA (viral or nanoparticle delivery)	Silence cGAS/STING to attenuate post-ischaemic inflammation and reduce infarct volume	Ischaemic stroke (MCAO/reperfusion, neonatal HIE, CVST)	Preclinical	[124,125,126]
LCP1	siRNA/shRNA (MoDM-targeted)	Silence LCP1 in MoDMs to reduce infarct and modulate immune response	Post-stroke immune response, neuroinflammation	Preclinical	[118]
IL-10	LNP-mRNA, Ad-IL-10, AAV-IL-10-MSCs	Upregulate IL-10 to enhance neuroprotection and reduce inflammation	Ischaemic stroke	Preclinical	[128,129,130]
IL-1β/TNF-α	AAV-shRNA	Silence pro-inflammatory mediators to attenuate neurotoxicity and tissue injury via C/EBPβ pathway	Ischaemic stroke	Preclinical	[131]
miR-124	EVs (microglia- or M2 macrophage-derived; sEVs)	miR-124 EVs suppress inflammation and glial scar, promote neurogenesis and repair	Stroke recovery	Preclinical	[134,135,136]
NeuroD1	AAV-NeuroD1 (GFAP::NeuroD1; FLEX systems)	In vivo glia-to-neuron conversion to rebuild local circuits	Neuroregeneration/reprogramming	Preclinical	[137,138,139,140]
Sirt1	CRISPR-dCas9-VP64 activation via CaP/PEI-PEG-bHb intranasal nanoparticles	Upregulate protective genes without altering DNA sequence	Ischaemic stroke (acute, permanent MCAO)	Preclinical	[141]
BDNF/TrkB	AAV-BDNF (intramuscular) + AAV-TrkB (intranasal)	Enhance CST connectivity and synaptic transmission to aid motor recovery	Angiogenesis & neurotrophic support	Preclinical	[143]
VEGF/VEGF-C	VEGF-modified BMSCs; AAV-VEGF-C (i.c.v., pre-treatment)	Promote angiogenesis, BBB protection, lymphatic drainage, neuroprotection	Vascular/BBB & lymphatic modulation	Preclinical	[144,145]

## Data Availability

No new data were created or analyzed in this study. Data sharing is not applicable.
